# Spirituality, religious affiliation, and health in Germany: a cross-sectional study including Bayesian network modeling

**DOI:** 10.3389/fmed.2026.1873209

**Published:** 2026-09-03

**Authors:** Christian S. Kessler, Michael Jeitler, Miriam Ortiz, Benno Brinkhaus, Maren M. Michaelsen, Julia K. Schiele, Florian Jeserich, Tido von Schoen-Angerer, Michael Teut, Manfred Wischnewsky

**Affiliations:** 1 Department of Integrative Health Care and Prevention, University of Augsburg and University Hospital Augsburg, Augsburg, Germany; 2 Department of Internal Medicine and Nature-Based Therapies, Immanuel Hospital Berlin, Berlin, Germany; 3 Institute of Social Medicine, Epidemiology and Health Economics, Charité - University Medical Center Berlin, Corporate Member of Freie Universität Berlin and Humboldt-Universität zu Berlin, Berlin, Germany; 4 Institute for General Practice and Interprofessional Care, University Hospital Tübingen, Tübingen, Germany; 5 Robert Bosch Center for Integrative Medicine and Health, Bosch Health Campus, Stuttgart, Germany; 6 Contilia Akademie, Essen, Germany; 7 Katholische Schule für Pflegeberufe Essen gGmbH, Essen, Germany; 8 Charité Competence Center for Traditional and Integrative Medicine, Charité - University Medical Center Berlin, Corporate Member of Freie Universität Berlin and Humboldt-Universität zu Berlin and Berlin Institute of Health, Berlin, Germany; 9 Multidisciplinary Center for Integrative Medicine, Geneva University Hospitals, Geneva, Switzerland; 10 Medizinische Hochschule Brandenburg Theodor Fontane, Brandenburg an der Havel, Germany; 11 Faculty of Mathematics and Computer Science, University of Bremen, Bremen, Germany

**Keywords:** Bayesian network, cross-sectional survey, Germany, health-related quality of life, online survey, religious affiliation, spirituality, TCIM

## Abstract

**Background:**

Spirituality has been proposed as a relevant dimension of health, yet its distribution in the general population and its associations with health-related factors remain insufficiently characterized. This study examined how self-reported spirituality and religious affiliation are distributed in Germany and how they are associated with chronic disease burden, health-related quality of life, nutrition-related attitudes, and attitudes toward traditional, complementary, and integrative medicine (TCIM).

**Methods:**

We conducted an anonymous cross-sectional online survey of adults living in Germany aged 18–75 years (*N* = 4,065). Self-reported spirituality and religious affiliation were assessed alongside sociodemographic variables, Sinus-Milieus^®^, health status, chronic health conditions, nutrition-related attitudes, TCIM attitudes, and health-related quality of life. Group differences were analyzed using chi-square and Wilcoxon tests. Multivariable regression models examined correlates of healthy nutrition and EQ-5D-5L. In addition, a Bayesian Network was used to explore conditional dependencies among 37 variables (*n* = 4,013).

**Results:**

Overall, 896 participants (22.0%) self-identified as spiritual, and 2,076 (51.1%) reported a religious affiliation. Women were overrepresented among spiritual participants (60.8%). Spirituality differed significantly by gender, age, Sinus-Milieus^®^, religious affiliation, overall health status, and health-related quality of life. Cross-classification of spirituality and religious affiliation yielded four groups: religious and spiritual (14.2%), spiritual but not religious (7.9%), religious but not spiritual (36.9%), and neither religious nor spiritual (41.1%). The proportion of participants identifying as spiritual increased with the number of chronic diseases. In multivariable analyses, spirituality, but not religious affiliation, was associated with healthy nutrition. In the multivariable EQ-5D-5L model, only overall health status and chronic disease burden remained significant.

**Conclusions:**

In this large German adult online sample, spirituality showed distinct socio-cultural patterning and was associated with nutrition-related attitudes, TCIM attitudes, and greater chronic disease burden. However, spirituality was not independently associated with health-related quality of life after adjustment for health status and chronic disease burden. Bayesian Network modeling offered an exploratory framework for examining these relationships. Further studies using more differentiated measures of spirituality and religion are needed in order to better understand, classify, and scientifically frame this complex field in a cross-disciplinary manner.

## Introduction

The relationship between spirituality and health care has received increasing scholarly and clinical attention over recent decades (1, 2). Spirituality is a multidimensional and contested construct. For the purposes of this study, we use an inclusive, person-defined understanding that encompasses experiences of meaning, purpose, connection, and transcendence and is not restricted to institutional religion (3, 4). Conceptually, spirituality may be understood either as a phenomenon outside or prior to formal religious commitment, or as an integral dimension of lived religious life. These understandings can coexist in plural societies and help explain why self-described spirituality and formal religious affiliation overlap only partly (5). This perspective aligns with broader person-centered models of health that seek to move beyond physiological dysfunction alone (6). The World Health Organization (WHO) defines health as a state of physical, mental, and social wellbeing rather than merely the absence of disease, and recent WHO-oriented frameworks have further emphasized wellbeing as a multidimensional construct, including the spiritual realm (7, 8). Against this background, spirituality has increasingly been discussed as a potentially relevant dimension of whole-person healthcare (4, 9–11).

Historically, medicine and spirituality were closely intertwined across cultures, while modern biomedical paradigms increasingly foregrounded measurable/scalable bodily processes and often relegated existential and spiritual concerns to the outer margins of formal (medical) care (12, 13). Revisiting spirituality in contemporary medicine therefore does not imply a return to premodern concepts of healing, esotericism or quackery; rather, it reflects an effort to better capture dimensions of illness experience that remain important to so many patients, particularly in the context of chronic and serious illness as well as of loss, dying and death (4). In clinical settings, spirituality may function as a resource for coping, orientation, adherence and meaning-making, while also remaining a domain in which distress or unmet needs become visible (4, 14–17). Complementary medical systems such as Ayurveda further illustrate that religious, spiritual, and medical dimensions may overlap historically and culturally, underscoring the need for careful conceptual differentiation (18).

Empirical literature links spirituality and spiritual care to mental health, coping, quality of life, and, in some studies, mortality (2, 4, 19). At the same time, much of this evidence is observational and therefore vulnerable to (substantial) confounding and reverse causation. Recent syntheses and policy-oriented work have accordingly called for more conceptually precise and methodologically robust research on spirituality as a determinant of health and wellbeing (4, 20). This need is especially relevant when spirituality is examined in general population samples rather than in narrowly defined clinical or religious subgroups.

These questions are particularly relevant in (Western) Europe, where declining church affiliation coexists with more individualized and plural forms of meaning-making (21, 22). In Germany, this changing landscape raises the question of whether spirituality and religious affiliation remain socially patterned and, if so, whether such patterning is captured adequately by conventional socioeconomic indicators alone. To address this, the present study draws not only on standard sociodemographic measures but also on the Sinus-Milieus^®^ model, which reflects broader socio-cultural lifeworlds and value orientations (23). This perspective may offer a more differentiated understanding of how spirituality is/may be embedded in contemporary German society and how it relates to health-relevant attitudes and practices, including nutrition-related orientations and attitudes toward Traditional, Complementary, and Integrative Medicine/Health (TCIM/TCIH).

Based on prior research, we expected spirituality and religious affiliation to show partly distinct sociodemographic and socio-cultural patterning; we also expected self-reported spirituality to be associated with health-related orientations such as nutrition and attitudes toward TCIM. Because associations between spirituality and health may reflect illness-related meaning-making, confounding, or reverse causation, we expected crude health-related associations to attenuate after multivariable adjustment. Against this theory-informed background, the present study examined spirituality as the primary construct of interest while treating religious affiliation as an important contextual and comparative variable. Specifically, we aimed to investigate (1) how self-reported spirituality and religious affiliation are distributed across sociodemographic characteristics and Sinus-Milieus^®^ in Germany, (2) how they are associated with chronic disease burden, health-related quality of life (EQ-5D-5L/EQ VAS), nutrition-related attitudes, and attitudes toward TCIM, and (3) whether Bayesian network modeling provides additional exploratory information on conditional dependencies among these variables (24–31). The Bayesian analysis was intended to complement, not replace, theory-informed conventional analyses. By integrating sociological and clinical perspectives, this study sought to provide a differentiated population-level account of spirituality in contemporary Germany.

## Methods

### Study design

This study was based on an anonymous, representative cross-sectional online survey of the German resident population aged 18–75 years. Further methodological details on survey development and sampling procedures have been reported previously (32). Data collection, processing, and storage were conducted in accordance with core ethical principles for research involving humans (Declaration of Helsinki) and quality and ethics standards applicable to online survey research (including voluntary participation, informed consent, confidentiality, and data minimization). The study was approved by the Ethics Committee of Charité – Universitätsmedizin Berlin and registered on ClinicalTrials.gov (NCT05530720).

The questionnaire was developed iteratively by the working group, with involvement of academic TCIM experts from German-speaking countries (one professor from Switzerland, six professors, and four postdoctoral senior researchers from Germany). The final questionnaire covered sociodemographic characteristics, TCIM use, attitudes toward spirituality, Sinus-Milieus^®^, health-related quality of life (EQ-5D-5L), and self-reported diseases/health conditions.

The survey was implemented by three German market research institutes (Conversio, Sinus, and Respondi) in close coordination with the study team. Conversio advised on survey methodology and feasibility for online implementation, coordinated collaboration with the other institutes, and supervised fieldwork. Sinus provided the Sinus-Milieus^®^ indicator. Respondi conducted the online survey and recruited respondents via its online access panel. Respondents remained anonymous to the research team.

Data collection was conducted using computer-assisted web interviewing (CAWI). The panel and survey process were reported as certified according to ISO 26362.

Eligibility criteria were provision of electronic informed consent, age ≥18 years, sufficient German language skills, and sufficient cognitive ability to participate in an approximately 30-min online survey (32). The exclusion criterion was lack of consent to participate.

### Outcomes

#### Spirituality and religious affiliation

Spirituality was assessed using a single-item self-rating question: “Would you describe yourself as spiritual?” (single answer). Response options were: Yes, very; Yes, somewhat; Neither; Rather not; Not at all; Don't know. No definition of “spiritual” was provided; respondents answered based on their own understanding. Religious affiliation was assessed separately by asking respondents whether they belonged to a religious community and, if so, which community. The full questionnaire (translated from German) is provided in Supplement S1.

#### Sociodemographic variables, health, and TCIM-related measures

Sociodemographic variables included age, sex/gender, education, income, and other standard indicators (see Supplement S1 for the full survey). Health-related quality of life was measured using the EQ-5D-5L. Self-reported diseases/conditions and TCIM-related variables (use and attitudes) were assessed via questionnaire items included in the underlying survey instrument described in Supplement S1 and the primary results publication (32).

#### Sociological segmentation: Sinus-Milieus^®^

To complement traditional sociodemographic indicators, the survey included the Sinus-Milieus^®^ classification provided by the Sinus Institute. The Sinus-Milieus^®^ are “groups of like-minded people” based on shared lifeworlds (values, fundamental orientations, and attitudes toward work, family, leisure, and consumption). The model is organized along two primary dimensions: social situation (vertical axis) and basic orientation (horizontal axis). Its inclusion allowed us to examine whether spirituality and religious affiliation vary not only by standard socioeconomic indicators but also by broader socio-cultural lifeworlds.

#### Statistical analysis

Binary, categorical, and ordinal variables are described using absolute numbers and percentages, and continuous variables by mean ± standard deviation or by median and quartiles, as appropriate. Group differences were assessed using Pearson's chi-square test or Wilcoxon's rank-sum test, as appropriate. Separate multivariable regression models were fitted for the perceived importance of healthy nutrition and for EQ-5D-5L. The covariates entered into the respective models are reported in the Results. All regression analyses were based on the complete cases available for the corresponding model.

#### Model construction and structure learning

We used a hybrid, theory-informed exploratory approach. First, an initial structure was specified from background knowledge and the temporal/logical ordering of variables where such ordering was defensible. Second, we compared multiple structure-learning algorithms, including Bayesian Search (27, 28), PC algorithm (29), Greedy Thick Thinning (GTT) (30), Tree Augmented Naïve Bayes (TAN) (31), Augmented Naïve Bayes (ANB) (31), and Naïve Bayes (NB) (31). Alternative structures were assessed for substantive plausibility, interpretability, and cross-validated predictive performance. Based on this comparative evaluation, GTT was selected for the final BN. Model learning and internal evaluation used 10-fold cross-validation. The conventional descriptive and multivariable models served as complementary robustness checks for the principal associations. Because observational cross-sectional data generally do not identify edge direction uniquely, directed edges were interpreted as a representation of the fitted joint probability structure and not as evidence of causal direction.

#### Bayesian network modeling

##### Rationale and variables

Bayesian networks (BNs) were used as an exploratory, interpretable probabilistic framework to examine conditional dependency structures among spirituality, religious affiliation, socio-cultural positioning (Sinus-Milieus^®^), and health-related variables collected in the survey. A BN represents variables as nodes in a directed acyclic graph (DAG) and encodes conditional dependencies in a joint probability distribution. In the present study, the BN complemented conventional analyses by depicting multivariable dependency structures and enabling probabilistic profiling based on the learned distribution. It was not used to estimate causal effects, and the learned directions should not be interpreted as demonstrating that spirituality causes milieu membership, health status, or any other included variable, or vice versa.

The set of variables entered into BN modeling comprised the survey measures relevant to the research questions, including spirituality self-identification, Sinus-Milieus^®^, sociodemographic variables, TCIM use/attitudes, health-related quality of life (EQ-5D-5L), and self-reported health conditions.

##### Software

Statistical analyses were performed using R (version 4.5.1; R Foundation) and IBM SPSS Statistics (version 31.0). Bayesian networks were calculated with GeNIe (version 5.0; BayesFusion LLC). The BN analysis was restricted to complete cases for the variables included in the network (*n* = 4,013); 52 cases with missing values in one or more BN variables were excluded from this part of the analysis.

## Results

## Sample characteristics, spirituality, and religious affiliation

Of the 4,065 participants aged 18–75 years, 51.7% were female. Mean (median) age was 49.3 ± 15.8 years (51.0 years). Educational attainment was distributed as follows: 42.5% with higher education (university degree or equivalent) and 56.8% with primary or secondary education. Approximately half (54.3%) reported a net household income between €2,000 and €5,000, with 7.5% earning above and 38.2% below this range. For detailed sociodemographic characteristics, please refer to the main publication (32).

Overall, 896 participants (22.0%) self-identified as “spiritual” and 2,076 (51.1%) reported a religious affiliation (Table 1). Among those who identified as spiritual, women were overrepresented (*n* = 545; 60.8%). Compared with non-spiritual participants, those who identified as spiritual were more likely to be female (*p* < 0.001), and spirituality varied significantly across age groups (*p* = 0.002) and Sinus-Milieus^®^ (*p* < 0.001). Spiritual participants were also more likely to report a religious affiliation (*p* < 0.001). Spirituality differed by overall health status (*p* = 0.010), though without a clear linear gradient. Spiritual participants had slightly lower EQ VAS scores (66.8 vs. 68.6; *p* = 0.030) and lower EQ-5D-5L index values (0.82 vs. 0.86; *p* < 0.001).

**Table 1:** Basic characteristics.

Characteristic	All (*n* = 4,065)	Are you spiritual?		*p*-value
		Yes (**n** = 896; 22%)	**No (*****n*** = **3,169; 78.0%)**	
	***n*** **(%)**	***n*** **(%)**	***n*** **(%)**	
Gender				
Male	1,947 (47.9)	345 (17.7)	1,602 (82.3)	< 0.001
Female	2,101 (51.7)	545 (25.9)	1,556 (74.1)	
15.6-7.2,-1.3499ptDiverse	17 (0.4)	6 (35.3)	11 (64.7)	
Age in years				
18 to 24	273 (6.7)	73 (26.7)	200 (73.3)	0.002
25 to 34	643 (15.8)	136 (21.2)	507 (78.8)	
35 to 44	672 (16.5)	144 (21.4)	528 (78.6)	
45 to 54	723 (17.8)	162 (22.4)	561 (77.6)	
55 to 64	888 (21.8)	227 (25.6)	661 (74.4)	
65 to 75	866 (21.3)	154 (17.8)	712 (82.2)	
Education				
No general school-leaving certificate (yet), still a pupil at a general school	30 (0.7)	7 (23.3)	23 (76.7)	0.231
Secondary (elementary, basic) school leaving certificate without completed apprenticeship/vocational training	251 (6.2)	61 (24.3)	190 (75.7)	
Secondary school leaving certificate with completed apprenticeship/vocational training	885 (21.8)	169 (19.1)	716 (80.9)	
Secondary school without A-levels (German: Realschulabschluss/Mittlere Reife/Oberschule) or equivalent qualification	1,173 (28.9)	254 (21.7)	919 (78.3)	
A-levels, (technical) university entrance qualification without studies	751 (18.5)	182 (24.2)	569 (75.8)	
Studies (university, college, university of applied sciences, polytechnic)	949 (23.3)	216 (22.8)	733 (77.2)	
PhD	26 (0.6)	7 (26.9)	19 (73.1)	
Net monthly household income				
Up to 1,000 €	505 (12.4)	133 (26.3)	372 (73.7)	0.071
1,000–2,000 €	1,047 (25.8)	244 (23.3)	803 (76.7)	
2,000–3,000 €	1,049 (25.8)	223 (21.3)	826 (78.7)	
3,000–4,000 €	735 (18.1)	148 (20.1)	587 (79.9)	
4,000–5,000 €	424 (10.4)	82 (19.3)	342 (80.7)	
> 5,000 €	305 (7.5)	66 (21.6)	239 (78.4)	
Sinus-Milieus^®^				
Conservative upscale Milieu	429 (10.6)	128 (29.8)	301 (70.2)	< 0.001
Post-materialist Milieu	575 (14.1)	140 (24.3)	435 (75.7)	
Performer Milieu	452 (11.1)	99 (21.9)	353 (78.1)	
Expeditive Milieu	417 (10.3)	66 (15.8)	351 (84.2)	
Adaptive-pragmatic middle class Milieu	475 (11.7)	104 (21.9)	371 (78.1)	
Nostalgic middle class Milieu	443 (10.9)	75 (16.9)	368 (83.1)	
Traditional Milieu	289 (7.1)	74 (25.6)	215 (74.4)	
Precarious Milieu	346 (8.5)	70 (20.2)	276 (79.8)	
Consumer-hedonistic Milieu	314 (7.7)	58 (18.5)	256 (81.5)	
Neo-ecological Milieu	325 (8.0)	82 (25.2)	243 (74.8)	
Religious community				
Catholic	853 (21.0)	240 (28.1)	613 (71.9)	< 0.001
Protestant	1,051 (25.9)	249 (23.7)	802 (76.3)	
Muslim	69 (1.7)	31 (44.9)	38 (55.1)	
Buddhist	17 (0.4)	13 (76.5)	4 (23.5)	
Hindu	2 (0.0)	1 (50.0)	1 (50.0)	
Jewish	12 (0.3)	7 (58.3)	5 (41.7)	
Other religious community	72 (1.8)	35 (48.6)	37 (51.4)	
No religious affiliation/atheist	1,989 (48.9)	320 (16.1)	1,669 (83.9)	
Religious affiliation				
Religious	2,076 (51.1)	576 (27.7)	1,500 (72.3)	< 0.001
No religious affiliation/atheist	1,989 (48.9)	320 (16.1)	1,669 (83.9)	
Overall health status				
Excellent	225 (5.5)	59 (26.2)	166 (73.8)	0.01
Very good	1,095 (26.9)	260 (23.7)	835 (76.3)	
Good	1,840 (45.3)	368 (20.0)	1,472 (80.0)	
Fair	766 (18.8)	186 (24.3)	580 (75.7)	
Bad	139 (3.4)	23 (16.5)	116 (83.5)	
EQ VAS, Mean (±SD)	68.2 (±22.3)	66.8 (±22.0)	68.6 (±22.4)	0.03
**EQ 5D-5L, Mean (±SD)**	0.85 (±0.21)	0.82 (±0.22)	0.86 (±0.20)	< 0.001

## Sociodemographic patterning and Sinus-Milieus^®^

Religious affiliation and spirituality showed partly different patterns across sociodemographic characteristics and Sinus-Milieus^®^. Religious affiliation differed significantly by educational attainment (*p* < 0.001). Specifically, 68% of individuals without a school-leaving certificate or with only primary education were religiously affiliated, compared to 57% of those with vocational certificates and 48% of those with higher education. In contrast, religious affiliation did not differ by net monthly household income (*p* = 1.0).

**Figure 1:**
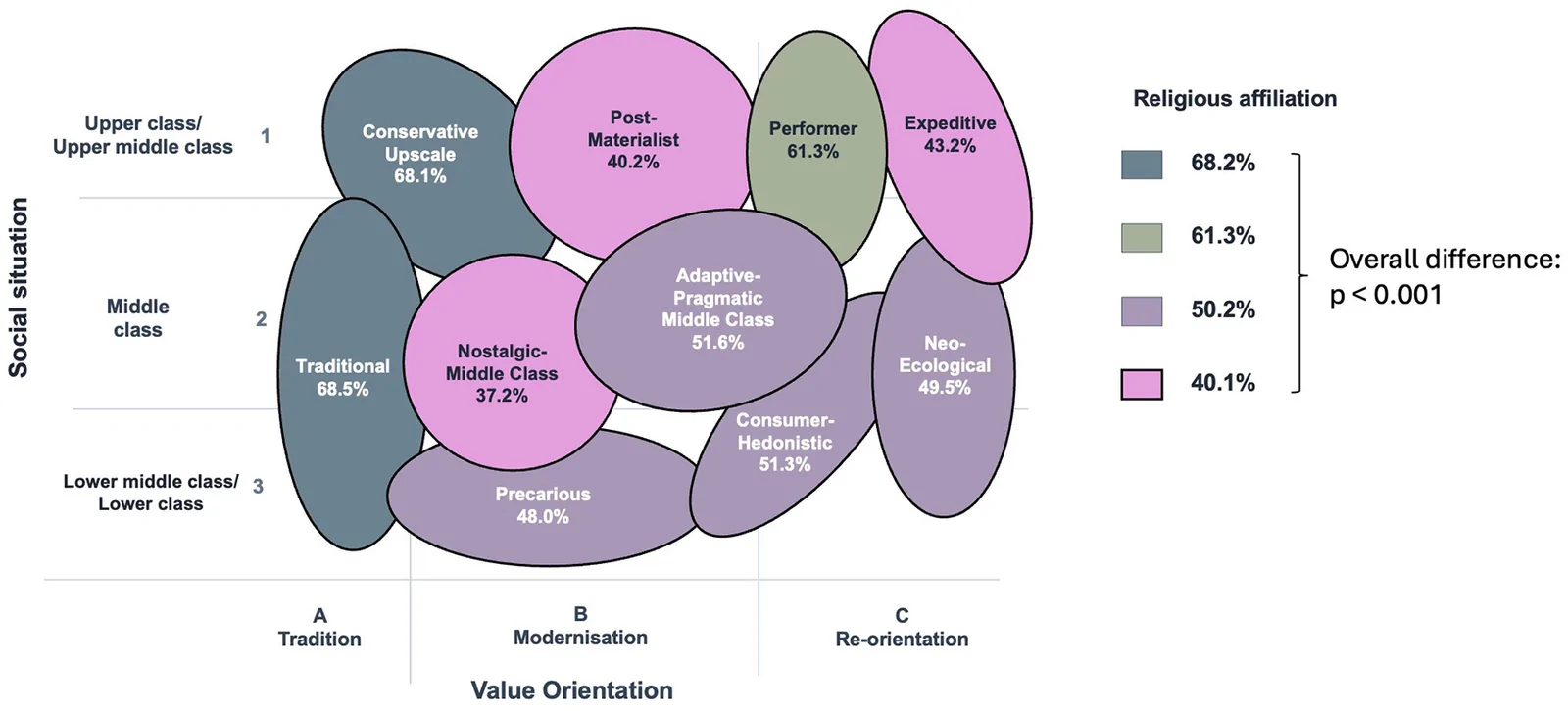
Religious affiliation across Sinus-Milieus^®^. Proportion of participants reporting a religious affiliation across Sinus-Milieus^®^. Overall group differences were significant (*p* < 0.001). Milieus sharing the same color did not differ significantly from one another.

Religious affiliation also differed across Sinus-Milieus^®^ (*p* < 0.001). The Conservative Upscale and Traditional milieus exhibited the highest religious affiliation (68.2%), whereas the Post-Materialists, the Nostalgic Middle Class, and the Expeditive milieu showed the lowest (40.1%) (Figure 1).

**Figure 2:**
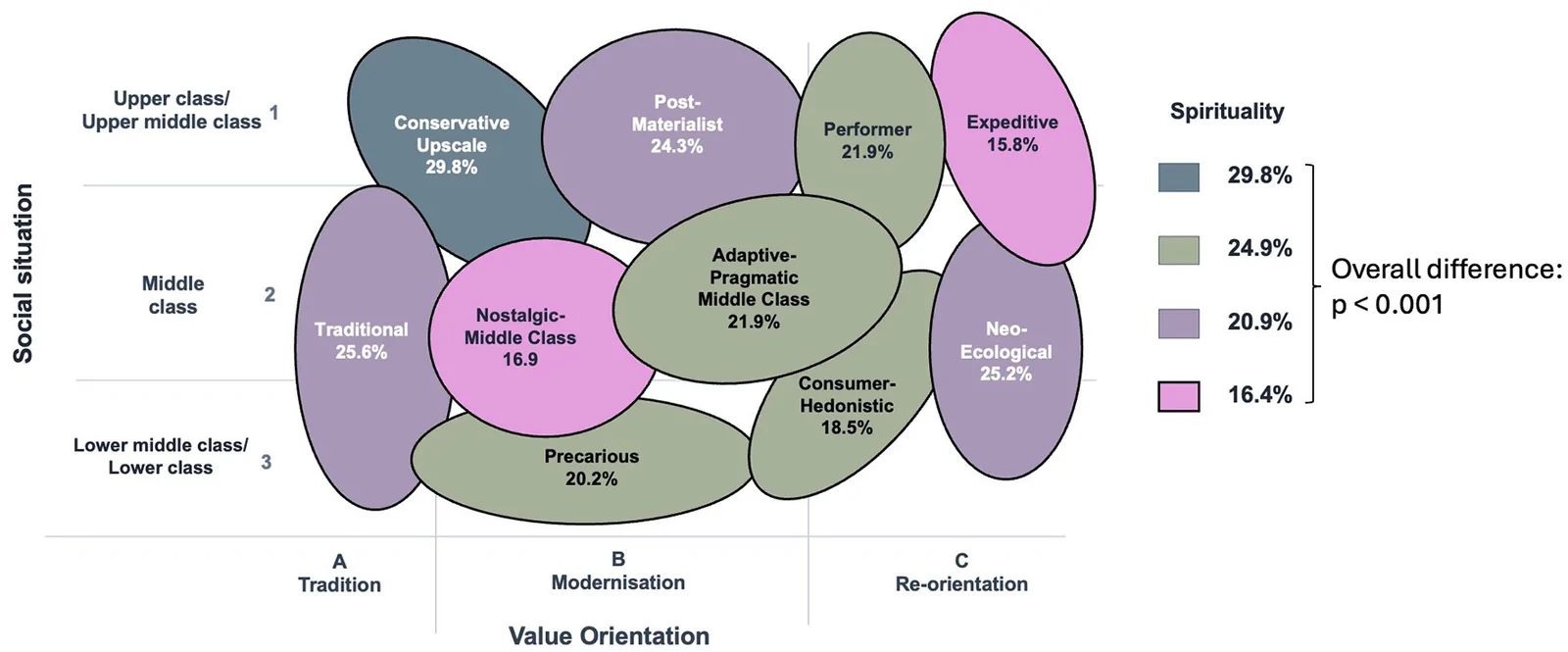
Spirituality across Sinus-Milieus^®^. Proportion of participants self-identifying as spiritual across Sinus-Milieus^®^. Overall group differences were significant (*p* < 0.001). Milieus sharing the same color did not differ significantly from one another.

**Figure 3:**
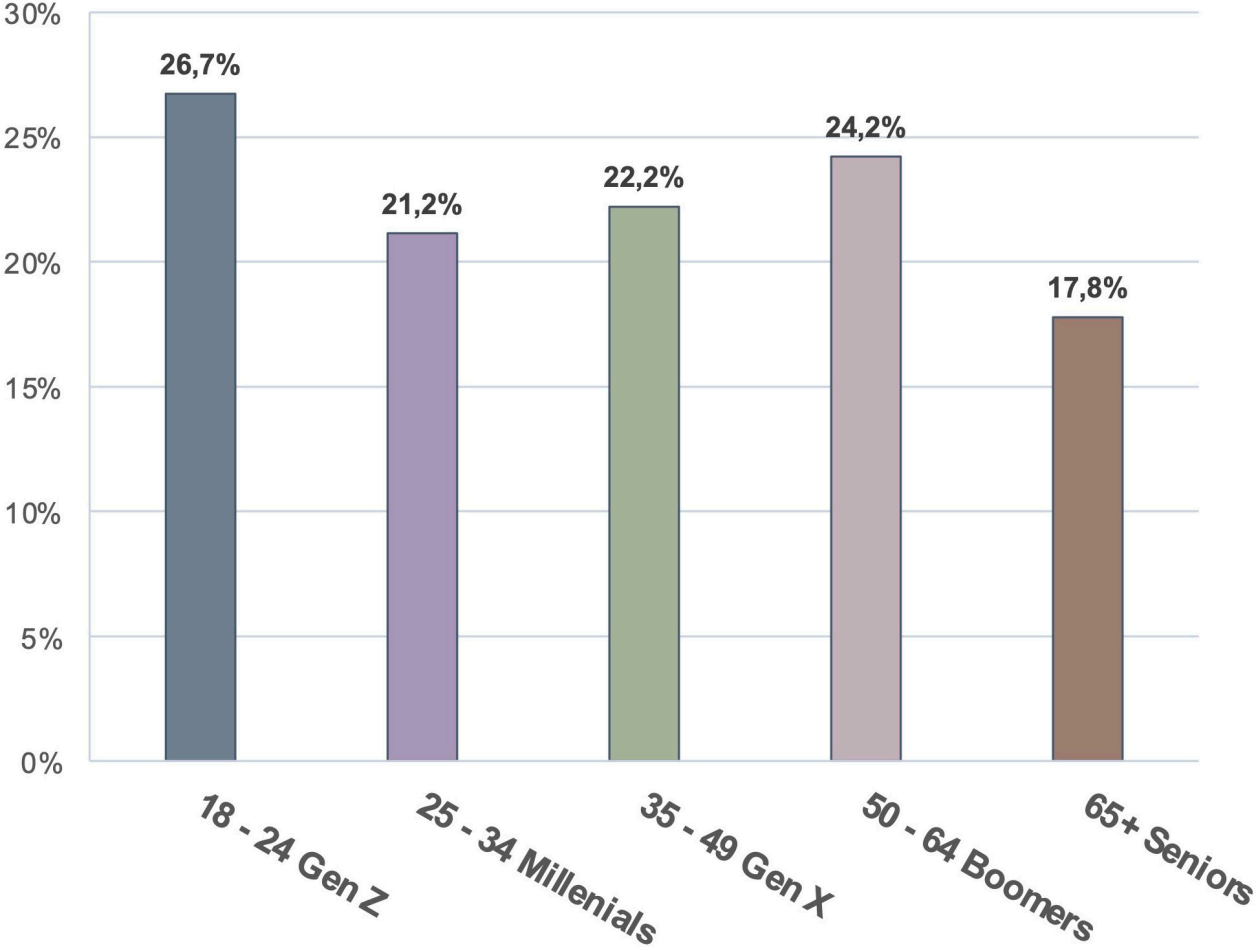
Spirituality across age groups. Proportion of participants self-identifying as spiritual across age groups. Overall group differences were significant (*p* = 0.002).

For spirituality, no significant differences were observed by educational attainment (*p* = 0.231) or net monthly household income (*p* = 0.071). However, spirituality differed significantly across Sinus-Milieus^®^(*p* < 0.001). The Conservative Upscale milieu exhibited the highest proportion of spirituality (29.8%), whereas the Nostalgic Middle Class and Expeditive milieus showed the lowest proportions (16.4%) (Figure 2). Spirituality also differed across age groups (*p* = 0.002), with the highest proportions among the youngest (18–24 years: 26.7%) and those aged 55–64 years (25.6%); the lowest proportion was observed among those aged 65–75 years (17.8%) (Table 1). A complementary stratification by generation is shown in Figure 3. Across milieus, spirituality also differed by gender (Table 2).

**Table 2:** Gender distribution among spiritual participants across Sinus-Milieus^®^.

Sinus-Milieus^^®^^	Gender			Sig.
	Female	Male	Diverse	
Conservative upscale	67.2%	32.8%		*p* = 0.014
Post-materialist	72.1%	27.9%		
Performer	54.5%	45.5%		
Expeditive	65.2%	33.3%	1.5%	
Adaptive-pragmatic	52.9%	47.1%		
Nostalgic middle class	62.7%	36.0%	1.3%	
Traditional	55.4%	44.6%		
Precarious	60.0%	40.0%		
Consumer-hedonistic	51.7%	44.8%	3.4%	
Neo-ecological	56.1%	41.5%	2.4%	

Gender distribution among participants who self-identified as spiritual, stratified by Sinus-Milieus^®^. Percentages are given by milieu. The overall association between gender and milieu among spiritual participants was significant (*p* = 0.014).

## Four descriptive spirituality/religious affiliation groups

Participants were classified into four descriptive groups based on religious affiliation (yes/no) and self-reported spirituality (yes/no): religious and spiritual (RAS), spiritual but not religious (SBNR), religious but not spiritual (RBNS), and neither religious nor spiritual (NRNS). The distribution across these groups was RAS 14.2%, SBNR 7.9%, RBNS 36.9%, and NRNS 41.1% (Table 3). These categories are intended as descriptive cross-classifications rather than fixed sociological archetypes.

**Table 3:** Four descriptive spirituality/religious affiliation groups in the study sample.

Archetype	Label	Formula	Description	Frequency
I	RAS	Religious affiliation: yes	Religious affiliation and self-reported spirituality	14.2%
		Self-reported spirituality: yes		
II	SBNR	Self-reported spirituality: yes	Self-reported spirituality without religious affiliation	7.9%
		Religious affiliation: no		
III	RBNS	Religious affiliation: yes	Religious affiliation without self-reported spirituality	36.9%
		Self-reported spirituality: no		
>IV	>NRNS	religious affiliation: no	Neither religious affiliation nor self-reported spirituality	41.1%
		Self-reported spirituality: no		

Participants were classified by cross-tabulation of religious affiliation (yes/no) and self-reported spirituality (yes/no) into four groups: religious and spiritual (RAS), spiritual but not religious (SBNR), religious but not spiritual (RBNS), and neither religious nor spiritual (NRNS). The table presents the classification rules, descriptive definitions, and group frequencies.

Within denominational groups, 71.9% of Catholics and 76.3% of Protestants were classified as RBNS. The four groups also differed significantly across aggregated Sinus main milieu categories (Supplementary Figure S1).

## Nutrition-related attitudes

Overall, 62.4% of participants considered nutrition important or very important. Nutrition-related attitudes differed significantly across the four groups (RAS, SBNR, RBNS, NRNS) (Supplementary Figure S2). In a multivariable model including both spirituality and religious affiliation, only spirituality emerged as a significant predictor (*p* < 0.001) of the perceived importance of healthy nutrition, whereas religious affiliation was not significant (*p* = 0.140). Healthy nutrition was considered important by 61.1% of the religiously unaffiliated and 63.5% of the religiously affiliated participants.

## TCIM attitudes and spirituality

In descriptive analyses, spirituality was associated with attitudes toward TCIM. Specifically, 78.3% of spiritual participants held a very or mostly positive view of TCIM, compared with 44.5% of non-spiritual participants.

## Chronic disease burden, health status, and health-related quality of life

**Figure 4:**
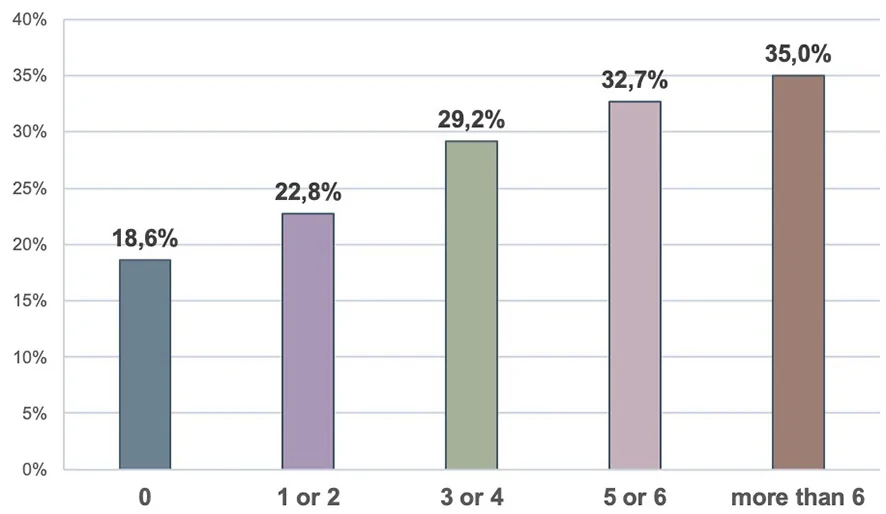
Spirituality by number of chronic diseases. Proportion of participants self-identifying as spiritual according to the number of self-reported chronic diseases. The proportion increased significantly with greater chronic disease burden (*p* < 0.001).

The proportion of participants identifying as spiritual increased significantly with the number of self-reported chronic diseases (*p* < 0.001; Figure 4). There was no significant difference in religious affiliation between participants with vs. without chronic diseases (*p* = 0.551).

For self-reported health, 32.5% rated their condition as “excellent” or “very good,” and 45.3% as “good.” Nonetheless, 71.1% reported some problems with mobility, self-care, daily activities, pain/discomfort, or anxiety/depression.

In descriptive analyses, spirituality was more frequent among individuals with general health problems than among those without (*p* < 0.001). The proportion of spiritual participants was also higher among those reporting severe problems in specific EQ-5D-5L domains, including severe anxiety/depression and severe pain/discomfort. A similar trend was observed for EQ-5D-5L scores, with worse health states associated with a higher proportion of spirituality (*p* < 0.001). Supplementary Figure S6 further illustrates differences in self-rated health by gender and spirituality status.

In a multivariable model for EQ-5D-5L including overall health status, spirituality, number of chronic diseases, gender, problems in EQ-5D-5L domains, net monthly household income, religious affiliation, and Sinus main milieus^®^, only overall health status and the number of chronic diseases remained statistically significant predictors of EQ-5D-5L (Supplementary Figure S3).

## Bayesian network findings

In addition to the descriptive and multivariable analyses reported above, Bayesian network modeling was used to explore conditional dependency structures across the included variables. The BN analysis was based on *n* = 4,013 participants and comprised 37 variables. Supplementary Figure S4 shows the final network learned with the GTT algorithm. The model includes gender, age, socioeconomic factors (income, education, and occupation), Sinus-Milieus^®^, religious affiliation, self-reported spirituality, attitudes toward TCIM and conventional medicine, importance of nutrition, health-related quality of life, and self-reported health conditions. The corresponding adjacency matrix is provided in Supplementary Table S1. Relative frequencies of the Sinus-Milieus^®^in the SINUS Institute reference distribution, the present study, and the Bayesian model are summarized in Supplementary Table S2. The network should be read as a compact representation of conditional dependencies across the full variable set. It does not resolve the direction of association between spirituality and Sinus-Milieus^®^or between spirituality and health, and it does not supersede the regression results. Rather, its main contribution is to show that spirituality is embedded in a broader configuration of religious affiliation, socio-cultural positioning, health-related attitudes, and morbidity-related variables.

## Profile of participants who identified as spiritual

BN-derived marginal probabilities for participants who identified as spiritual indicated that 18% were “very spiritual” and 81% “somewhat spiritual.” A majority (64%) reported a religious affiliation, most commonly Protestant (28%), Catholic (27%), or Muslim (4%), while all other religious communities each accounted for less than 1%. Regarding health-related attitudes, 79% of spiritual participants held a “very” or “mostly” positive view of TCIM. Attitudes toward conventional medicine were more heterogeneous: 48% reported a “mostly positive” and 13% a “very positive” attitude, whereas 6% and 2% reported “mostly negative” and “very negative” attitudes, respectively. In terms of lifestyle, 78% considered nutrition important or very important. Regarding self-reported health, 35% rated their condition as “excellent” or “very good,” and 41% as “good.” Nevertheless, 78% reported at least some problems in one or more EQ-5D-5L domains (Supplementary Figure S5).

## Discussion

In this population-based survey of adults in Germany, spirituality—considered here as the primary construct of interest and interpreted alongside religious affiliation—remained a socially patterned and clinically relevant construct despite declining institutional religious affiliation (in Germany). Self-reported spirituality differed by gender, age, and Sinus-Milieus^®^, was consistently associated with nutrition-related attitudes and TCIM acceptance, and was more frequent among participants with a higher burden of chronic disease. At the same time, spirituality was not independently associated with health-related quality of life once overall health status and chronic disease burden were taken into account. Taken together, these findings suggest that spirituality in this sample was more closely related to socio-cultural positioning, nutrition-related attitudes, and TCIM attitudes than to health-related quality of life after adjustment.

## Socio-cultural patterning beyond socioeconomic status

A key contribution of this study is the use of Sinus-Milieus^®^ to move beyond conventional socioeconomic indicators. Education and income were only partially informative for religious affiliation and spirituality, whereas the milieu model showed clearer differentiation in both domains. However, these associations require cautious interpretation. The Sinus classification itself incorporates values and orientations that may overlap conceptually with how respondents understand spirituality; spirituality may also shape lifestyle and value orientation. Consequently, the observed milieu differences are potentially endogenous and cannot be interpreted as effects of milieu membership on spirituality, or of spirituality on milieu assignment. In this cross-sectional study, the milieu findings are descriptive evidence of socio-cultural co-patterning rather than causal relationships.

## A four-group framework highlights heterogeneity

The four-group classification (RAS, SBNR, RBNS, NRNS) further underscores that a simple religious-versus-secular distinction is insufficient. The SBNR and NRNS groups show that non-affiliation does not imply uniform secularity, whereas the RBNS group indicates that religious affiliation may persist without self-identification as spiritual. This pattern is compatible with two conceptually different uses of spirituality: as an individualized or pre-institutional phenomenon that may exist outside religion, and as a dimension of lived religious life (5). The meaning of the term is therefore likely to vary across respondents; in some institutionally religious groups, “spirituality” may be experienced as integral to religion, while in others it may be understood as distinct from or even in tension with organized religion. The four groups should consequently be interpreted as descriptive cross-classifications rather than fixed sociological types.

## Clinical relevance: spirituality as a potential resource in whole-person care

From a clinical perspective, our findings are consistent with spirituality becoming more salient in the context of illness, but they do not demonstrate that spirituality is uniformly beneficial. Religious and spiritual coping can include positive forms, such as meaning-making, collaborative problem-solving, hope, forgiveness, and support from a community, as well as negative forms, such as spiritual struggle, perceived abandonment, guilt, or conflict with a religious community (19, 33, 34). These forms may coexist and may relate differently to wellbeing. Because our survey assessed only self-described spirituality and religious affiliation, it could not distinguish coping styles, spiritual practices, or spiritual distress. The descriptive increase in spirituality with chronic disease burden may therefore reflect resource mobilization, illness-related searching and reappraisal, distress, reverse causation, or combinations of these processes. The adjusted EQ-5D-5L model further indicates that spirituality was not independently associated with health-related quality of life after accounting for overall health status and chronic disease burden.

This has practical implications for person-centered and individualized (medical) care. When clinically appropriate and led by the patient's preferences, brief spiritual assessment may help identify sources of meaning, connection, and support that shape coping and decision-making. Structured tools such as the FICA spiritual history tool and the HOPE questions provide pragmatic, non-prescriptive approaches for spiritual history-taking (14, 35, 36), particularly in settings such as oncology, palliative care, geriatrics, and mental health, where existential concerns often accompany symptom burden (4). Any spiritually sensitive communication should remain ethically grounded, avoid proselytizing, and always be guided by the patient's own language and priorities (4).

## Spirituality within broader health-related orientations

The observed links between spirituality, nutrition-related attitudes, and TCIM acceptance suggest that spirituality may be embedded in broader health-related orientations rather than in isolated beliefs or practices. In this sense, spirituality may function less as a single explanatory variable than as part of a wider orientation toward health, self-care, and meaning. This interpretation is compatible with pluralistic and person-centered models of health that acknowledge existential dimensions alongside biological, psychological, and social ones (8, 37, 38). Research on placebo/nocebo, nonspecific effects and meaning responses also supports the view that meaning attribution, therapeutic context, and relational factors can influence symptoms and outcomes through plausible psychobiological pathways (39, 40).

## Methodological contribution of Bayesian networks

Bayesian network modeling added an exploratory methodological perspective by representing conditional dependency structures across spirituality, religious affiliation, socio-cultural positioning, and health-related variables within one probabilistic framework (26). The analysis was theory-informed, compared several candidate algorithms, and used 10-fold cross-validation; it was therefore not intended as an unconstrained substitute for theoretical reasoning. Its contribution was to situate spirituality within a multivariable configuration and to generate conditional probability profiles that complement the descriptive and regression analyses. Nevertheless, the BN does not solve confounding, reverse causation, measurement overlap, or the endogeneity of spirituality and Sinus-Milieus^®^. Edge directions in a cross-sectional learned network are not causal findings, and equivalent or near-equivalent structures may fit the data. The BN results should therefore be regarded as hypothesis-generating and interpreted only in conjunction with the conventional analyses and the study's substantive framework (26, 29).

## Strengths and limitations

This study has several strengths, including the large, online-representative sample, the combination of health-related variables with socio-cultural segmentation via Sinus-Milieus^®^, and the use of Bayesian network modeling as an additional exploratory analytic framework. Several limitations should also be considered. First, the cross-sectional design precludes causal inference and leaves open confounding, endogeneity, and reverse causation, for example if illness increases spiritual self-identification or if spirituality and milieu classification partly reflect overlapping value orientations. Second, spirituality was measured with a single self-report item without an explicit definition. This pragmatic measure captured participants' self-identification but not the multidimensional content, intensity, practices, coping functions, or possible distress associated with spirituality. Third, religious affiliation should not be equated with religiosity or religious coping; the survey captured institutional affiliation more directly than lived religious practice, intensity, belief, positive coping, negative coping, or spiritual struggle. Fourth, country of birth, migration background, and duration of residence in Germany were not assessed. We could therefore not examine culturally shaped variation associated with migration histories. Fifth, although the online panel followed established quality standards, findings from a German-language panel may not generalize to people with limited German proficiency, non-panel populations, other countries, or cultural settings in which spirituality and religion are conceptualized differently. Sixth, participants may have differed in their ability to distinguish spirituality from religion because these terms overlap in everyday language. Finally, although Bayesian networks offer interpretable representations of conditional dependencies, they do not in themselves provide evidence of causal effects, and their structure may be sensitive to modeling choices (26, 29).

## Future research

Future research should build on these findings with longitudinal, mixed-methods, interdisciplinary, and intervention designs to clarify whether spirituality precedes, follows, or changes together with health-related experiences and behaviors. More differentiated measurement should distinguish spirituality outside formal religion from spirituality as lived within religious traditions and should assess positive and negative religious/spiritual coping, practice, communal participation, spiritual distress, and metaphysical beliefs or orientations. Studies should also collect country of birth, migration history, language, and duration of residence where ethically and methodologically appropriate. Cross-cultural comparisons are especially important because the meanings, social desirability, and health-related functions of spirituality and religious affiliation differ across cultural and religious contexts. Such work could test whether the German patterns observed here replicate elsewhere and could inform ethically robust approaches to spiritual history-taking, referral pathways, and professional training in spiritually sensitive care (14, 35, 36).

## Conclusion

This study provides population-level evidence on spirituality, religious affiliation, and health-related domains in a large German adult sample and shows how probabilistic modeling can be used to examine complex interdependencies. Self-reported spirituality showed distinct patterns across gender, age, and socio-cultural milieus, was associated with nutrition-related attitudes and TCIM acceptance, and was more frequent among participants with higher chronic disease burden. However, spirituality did not remain an independent predictor of health-related quality of life in models that accounted for overall health status and chronic disease burden.

The health-related implications of spirituality and religion represent an important interdisciplinary field of research with considerable clinical relevance, yet numerous questions remain insufficiently addressed or unanswered.

Future research should extend these findings with longitudinal designs and with richer measures of spirituality and religion, including spiritual coping, spiritual/religious practice, communal participation, spiritual distress, and metaphysical beliefs or orientations. Cross-cultural and implementation research will be important to test generalizability and to develop ethically grounded models of spiritually sensitive care.

## Data Availability

The dataset on which this study is based is not readily available because analyzes and publications based on this dataset are still ongoing. Access may be granted upon justified request and directed to michael.jeitler@charite.de.

## In memoriam

The authors dedicate this article to Boike Rehbein (†11.6.2022). Boike Rehbein was involved in the planning and initiation of the project and sadly passed away during the project.
