# Supplementary material for: Spirituality, religious affiliation, and health in Germany: a cross-sectional study including Bayesian network modeling
Source: Front Med (Lausanne). 2026 Sep 3;13:1873209. doi: 10.3389/fmed.2026.1873209 (PMC13581794; doi:10.3389/fmed.2026.1873209)
Supplement: Supplementary file 1 [file Table_1.docx]

**Supplementary Tables**

**Supplementary Table S1. Adjacency matrix of the Bayesian network. Adjacency matrix corresponding to the Bayesian network shown in Supplementary Figure S4. Matrix entries indicate the presence of conditional dependencies between variables in the final network structure. Variable labels reflect the coding used in the BN software; in some cases, related constructs appear in different coded forms (e.g., dichotomized vs. graded variables).**

|  | Acute gastrointestinal illnesses | Acute respiratory illnesses | Age groups | Allergies | Attitude towards TCIM | Attitude towards conventional medicine | Cancer | Cardiovascular diseases | Chronic gastrointestinal diseases | Chronic respiratory diseases | Diabetes mellitus | EQ 5D-5L anxiety/depression | EQ 5D-5L any problems | EQ 5D-5L mobility | EQ 5D-5L pain/discomfort | EQ 5D-5L self care | EQ 5D-5L usual activities | Education | Gender | General_Health_4grps | Headache_disorders | Importance of nutrition | Mental illnesses | Neurological diseases | Others | Painful disorders of the musculoskeletal system | Religious affiliation | Religious community | Sinus® Main Milieus | Sinus® Milieus | Skin diseases | Spiritual | Spirituality | Thyroid diseases | Total income | Use of TCIM within the last 12 mths | Your nutrition |
| --- | --- | --- | --- | --- | --- | --- | --- | --- | --- | --- | --- | --- | --- | --- | --- | --- | --- | --- | --- | --- | --- | --- | --- | --- | --- | --- | --- | --- | --- | --- | --- | --- | --- | --- | --- | --- | --- |
| Acute gastrointestinal illnesses |  |  |  |  |  |  |  |  |  |  |  |  |  |  |  |  |  |  |  |  |  |  |  |  |  |  |  |  |  |  |  |  |  |  |  |  |  |
| Acute respiratory illnesses | X |  |  |  |  |  |  |  |  |  |  |  |  |  |  |  |  |  |  | X |  |  |  |  |  |  |  |  |  |  |  |  |  |  |  |  |  |
| Age groups |  |  |  |  |  | X | X |  |  | X |  |  |  |  |  |  |  |  |  |  |  |  |  |  | X |  |  |  |  |  |  |  |  |  |  |  |  |
| Allergies |  | X |  |  |  |  |  |  |  | X |  |  |  |  |  |  |  |  |  |  |  |  |  |  |  |  |  |  |  |  |  |  |  |  |  |  |  |
| Attitude towards TCIM |  |  |  |  |  |  |  |  |  |  |  |  |  |  |  |  |  |  |  |  | X |  |  |  |  |  |  |  |  |  |  |  |  |  | X |  |  |
| Attitude towards conventional medicine |  |  |  |  | X |  |  |  |  |  |  |  |  |  |  |  |  |  |  |  |  |  |  |  |  |  |  |  |  |  |  |  |  |  |  |  |  |
| Cancer |  |  |  |  |  |  |  |  |  |  |  |  |  |  |  |  |  |  |  |  |  |  |  |  |  |  |  |  |  |  |  |  |  |  |  |  |  |
| Cardiovascular diseases |  |  |  |  |  |  |  |  |  | X |  |  |  |  |  |  |  |  |  |  |  |  |  |  |  |  |  |  |  |  |  |  |  | X |  |  |  |
| Chronic gastrointestinal diseases | X |  |  |  |  |  |  |  |  |  |  |  |  |  |  |  |  |  |  |  |  |  |  |  |  |  |  |  |  |  |  |  |  |  |  |  |  |
| Chronic respiratory diseases |  | X |  |  |  |  |  |  |  |  |  |  |  |  |  |  |  |  |  |  |  |  |  |  |  |  |  |  |  |  |  |  |  |  |  |  |  |
| Diabetes mellitus |  |  |  |  |  |  |  |  |  |  |  |  |  |  |  |  |  |  |  |  |  |  |  |  |  |  |  |  |  |  |  |  |  |  |  |  |  |
| EQ 5D-5L anxiety/depression |  |  |  |  |  |  |  |  |  |  |  |  |  |  |  |  |  |  |  |  |  | X |  |  |  |  |  |  |  |  |  |  |  |  |  |  |  |
| EQ 5D-5L any problems |  |  |  |  |  |  |  |  |  |  |  |  | X |  |  | X |  |  |  |  |  |  |  | X |  |  |  |  |  |  |  |  |  |  |  |  |  |
| EQ 5D-5L mobility |  |  |  |  |  |  |  |  |  |  |  |  |  | X |  | X | X |  |  |  |  |  |  |  |  |  |  |  |  |  |  |  |  |  |  |  |  |
| EQ 5D-5L pain/discomfort |  |  |  |  |  |  |  | X |  |  |  | X | X | X |  |  |  |  | X |  |  |  |  | X |  |  |  |  |  |  |  |  |  |  |  |  |  |
| EQ 5D-5L self care |  |  |  |  |  |  |  |  |  |  |  |  |  |  |  |  | X |  |  |  |  |  |  |  |  |  |  |  |  |  |  |  |  |  |  |  |  |
| EQ 5D-5L usual activities |  |  |  |  |  |  |  |  |  |  |  |  |  |  | X |  |  | X |  |  |  | X |  |  |  |  |  |  |  |  |  |  |  |  |  |  |  |
| Education |  |  | X |  |  |  |  |  |  |  |  |  |  |  |  |  |  |  |  |  |  |  |  |  |  |  |  |  |  |  |  |  | X |  |  |  |  |
| Gender |  |  |  |  |  |  |  |  |  |  |  |  |  |  |  |  |  |  |  | X |  |  |  |  |  |  |  |  |  |  |  | X |  |  |  |  |  |
| General health |  |  |  |  |  |  |  |  |  |  | X |  |  |  |  |  |  |  |  |  |  |  |  |  |  |  |  |  |  |  |  |  |  |  |  |  |  |
| Headache disorders |  |  |  |  |  |  |  |  |  |  |  |  |  |  |  |  |  |  |  |  |  |  |  |  |  |  |  |  |  |  |  |  |  |  |  |  |  |
| Importance of nutrition |  |  |  |  |  |  |  |  |  |  |  |  |  |  |  |  |  |  |  |  |  |  |  |  |  |  | X |  |  |  |  |  |  |  | X |  |  |
| Mental illnesses |  |  |  |  |  |  |  | X |  |  |  |  |  |  | X |  |  | X |  |  |  |  |  | X |  |  |  |  |  |  |  |  |  |  |  |  |  |
| Neurological diseases |  |  |  |  |  |  |  |  |  |  |  |  |  |  |  |  |  |  |  |  |  |  |  |  |  |  |  |  |  |  |  |  |  |  |  |  |  |
| Others |  |  |  |  |  |  |  |  |  |  |  |  |  |  |  |  |  |  |  |  |  |  |  |  |  |  |  |  |  |  |  |  |  |  |  |  |  |
| Painful disorders of the musculoskeletal system |  |  |  |  |  |  |  | X |  |  |  |  |  |  |  |  |  |  |  |  |  |  | X |  |  |  |  | X |  |  | X |  |  |  |  |  |  |
| Religious affiliation |  |  |  |  |  |  |  |  |  |  |  |  |  |  |  |  |  |  |  |  |  |  |  |  |  |  | X |  |  |  |  |  |  |  |  |  |  |
| Religious community |  |  |  |  |  |  |  |  |  |  |  |  |  |  |  |  |  |  |  |  |  |  |  |  |  |  |  |  |  |  |  |  |  |  |  |  |  |
| Sinus® Main Milieus |  |  |  |  |  |  |  |  |  |  |  |  |  |  |  |  |  |  |  |  |  |  |  |  |  |  |  |  |  |  | X |  |  |  |  |  |  |
| Sinus® Milieus |  |  |  |  |  |  |  |  |  |  |  |  |  |  |  |  |  |  | X |  |  |  |  |  |  |  | X |  |  |  |  |  |  |  |  |  |  |
| Skin diseases |  |  | X |  |  |  |  |  |  |  |  |  |  |  |  |  |  |  |  |  |  |  |  |  |  |  |  |  |  |  |  |  |  |  |  |  |  |
| Spiritual | X | X | X | X | X | X | X | X | X | X | X | X | X | X | X | X | X | X | X | X | X | X | X | X | X | X | X | X | X | X | X | X | X | X | X | X | X |
| Spirituality |  |  |  |  | X |  |  |  |  |  |  |  |  |  |  |  |  |  |  |  |  |  |  | X |  |  | X | X | X | X |  |  |  | X |  |  |  |
| Thyroid diseases |  |  |  |  |  |  |  |  |  |  |  |  |  |  |  |  |  |  |  |  |  |  |  |  |  |  |  |  |  |  |  |  |  |  |  |  |  |
| Total income |  |  |  |  |  |  |  |  |  |  |  |  |  |  |  |  |  |  |  |  |  |  |  |  |  |  |  |  |  |  |  |  |  |  |  |  |  |
| Use of TCIM within the last 12 mths |  | X | X |  |  |  |  |  |  |  |  |  |  |  |  |  |  |  |  |  |  |  |  |  |  |  |  |  |  |  |  |  |  |  |  |  |  |
| Nutrition |  |  |  |  |  |  |  |  |  |  |  |  |  |  |  |  |  | X |  |  |  |  |  |  |  |  |  |  |  |  |  |  |  |  |  |  |  |

**Supplementary Table S2. Relative frequencies and characteristics of the Sinus-Milieus® in the reference distribution, study sample, and Bayesian network sample**. Comparison of Sinus-Milieus® frequencies from the SINUS Institute reference distribution (Germany 2021), the present study sample, and the Bayesian network sample. The table also summarizes social status, value orientation, and short milieu descriptions.

| **Milieu** | **SINUS Institute (Germany 2021)** | **Study results** | **Bayesian model** | **Social Status** | **Value Orientation** | **Short Description** |
| --- | --- | --- | --- | --- | --- | --- |
| Conservative Upscale | 11% | 10.6% | 11% | High | Traditional | The structural conservative elite; ethics of responsibility, status, order. |
| Post-Materialist | 12% | 14.1% | 14% | High | Modern/Re-orientation | The educated sustainability elite; common good, diversity, self-determination. |
| Performer | 10% | 11.1% | 11% | High | Modernization | The efficiency elite; global economic thinking, tech-savvy, competitive. |
| Expeditive | 10% | 10.3% | 10% | High | Re-orientation | The creative avant-garde; urban, hip, digital, networked, unconventional. |
| Neo-Ecological | 8% | 8.0% | 8% | High/Middle | Re-orientation | The drivers of transformation; optimism, pragmatism, party & protest. |
| Adaptive-Pragmatic | 12% | 11.7% | 12% | Middle | Modernization | The modern mainstream; flexibility, utility, desire for belonging. |
| Nostalgic Middle Class | 11% | 10.9% | 11% | Middle | Traditional | The anxious center; longing for the past, fear of decline, loss of control. |
| Consumer-Hedonistic | 8% | 7.7% | 7% | Low-Middle | Modern/Re-orientation | The fun-oriented lower middle; entertainment, escapism, brand-focused. |
| Traditional | 10% | 7.1% | 7% | Lower | Traditional | The security-loving older generation; thrift, modesty, adaptation. |
| Precarious | 9% | 8.5% | 9% | Lower | Re-orientation (struggling) | The excluded; struggle for participation, resentment, resignation. |
